# Rethinking Strategies for Positive Newborn Screening Result (NBS+) Delivery (ReSPoND): a process evaluation of co-designing interventions to minimise impact on parental emotional well-being and stress

**DOI:** 10.1186/s40814-019-0487-5

**Published:** 2019-09-04

**Authors:** Jane Chudleigh, Jim Bonham, Mandy Bryon, Jill Francis, Louise Moody, Steve Morris, Alan Simpson, Fiona Ulph, Kevin Southern

**Affiliations:** 1 Centre for Maternal and Child Health Research, City, University of London, London, EC1V 0HB UK; 2 Division of Pharmacy, Diagnostics and Genetics Sheffield Children’s NHS Foundation Trust, Sheffield, S10 2TH UK; 3 Paediatric Psychology and Play Services, Great Ormond Street Hospital for Children NHS Foundation Trust, London, WC1N 3JH UK; 4 Division of Health Services Research and Management, City, University of London, London, EC1V 0HB UK; 5 School of Art and Design, Coventry University, Coventry, CV1 5FB UK; 6 Centre of Applied Health Research, University College London, London, WC1E 6BT UK; 7 Centre for Mental Health Research, City, University of London, London, EC1V 0HB UK; 8 Division of Psychology and Mental Health, The University of Manchester, Manchester, M13 9PL UK; 9 Women’s and Children’s Health, University of Liverpool, Liverpool, L69 3BX UK

**Keywords:** Newborn bloodspot screening, Cystic fibrosis, Sickle cell disease, Metabolic, Congenital hypothyroid

## Abstract

**Background:**

Newborn blood spot (NBS) screening seeks to prevent ill health, disability and death through early diagnosis and effective intervention. Each year, around 10,000 parents of babies born in England are given a positive NBS result indicating their child may be affected or carriers of one of the nine conditions currently screened for. Despite guidance, these results are inconsistently delivered to parents across geographical regions. There is evidence that many parents are dissatisfied with how NBS results are communicated to them and that poor communication practices can lead to various negative sequelae. The purpose of this study is to co-design, implement and undertake a process evaluation of new, co-designed interventions to improve delivery of initial positive NBS results to parents.

**Methods:**

This mixed-methods study will use four phases with defined outputs. Family Systems Theory will form the theoretical basis for the study. The principles and methods of experience-based co-design will underpin intervention development. Normalisation Process Theory will underpin the process evaluation of the interventions co-designed to improve the delivery of positive NBS results to parents. An economic analysis will determine resource use and costs of current practice and of implementing the new co-designed interventions. The nominal group technique will be used to inform the selection of suitable outcome measures for a future evaluation study.

**Discussion:**

The main output of the proposed study will be co-designed interventions for initial communication of positive NBS results to parents ready to be evaluated in a definitive evaluation study.

The interventions, co-designed with parents, will help to minimise potential negative sequelae associated with poor communication practices by considering parental and staff experiences as well as healthcare challenges such as finite resources. In addition, information about indicative costs associated with different communication strategies will be determined.

It is anticipated it may also be possible to extrapolate principles of good communication practices from the present study for the delivery of bad news to parents for children newly diagnosed with other conditions including cancer and other chronic conditions such as diabetes or epilepsy.

**Trial registration:**

ISRCTN 15330120 date of registration 17/01/2018

## Background

Newborn bloodspot screening (NBS) in the UK involves obtaining parents’ informed consent to take a small sample of blood from their baby’s heel (heel prick) on day 5 of life to determine if the baby may be affected by one of nine life-changing conditions^1^. These conditions are as follows: sickle cell disease (SCD), cystic fibrosis (CF), congenital hypothyroidism, phenylketonuria, medium-chain acyl-CoA dehydrogenase deficiency, maple syrup urine disease, isovaleric acidaemia, glutaric aciduria type 1 and homocystinuria (pyridoxine unresponsive). A positive NBS (NBS+) result indicates that the baby may be affected or be a carrier of one of these conditions and will often require further diagnostic testing before a definitive diagnosis is made [1].

Each year in England, over 10,000 parents are informed of their child’s NBS+ result around 2–8 weeks, depending on the condition, after birth [2, 3]. Most babies with initial NBS+ results for SCD and approximately half of those with an NBS+ result for CF will later be confirmed as gene carriers but unaffected by the disease. However, over 1300 babies will eventually be diagnosed as being affected by one of the conditions currently screened for [2, 3].

### Variation in communication practice in the UK

There is evidence of regional variations in the UK with regard to the approaches used to communicate NBS+ results and, in particular, suspected carrier status for CF and SCD following NBS. These approaches include receiving the result by letter to in-person communication during a home visit [4, 5]. The findings of Kai et al.’s study [5] informed the development of the current national guidelines for the communication process in the NBS Programme (NBSP), which recommend face-to-face communication by an appropriately trained health professional [1]. Despite these guidelines, a recent study with parents exploring their experience of receiving CF or SCD carrier results following NBS indicated that disparity continues to exist regarding how the guidelines are implemented in practice [6].

### Impact of poor communication practices

Poor, or inappropriate, communication strategies for NBS+ can influence parental outcomes in the short term [6–11] and may also have long-term impact [12]. Evidence suggests the distress caused can manifest in several ways including arguments between couples including apportioning of blame [6, 9, 13], alteration of life plans and inability to conduct tasks of daily living such as going to work or socialising [6], long-term alterations in parent-child relationships [12] and mistrust and lack of confidence affecting ongoing relationships with staff [9]. There is also evidence of increased parental distress resulting in parents reducing their child’s interaction with others, particularly in the case of CF [6]. Parents also experienced poor intra- and interpersonal relationships within their family system and more widely [14].

With the expansion of the NBSP in the UK in 2015 and further future growth planned [15], it is timely to ensure that clinical advantages of this process continue to outweigh potential long term negative psychosocial consequences for the families involved. It is essential that approaches used to deliver this information to parents are informed by them and shaped to meet their needs. It may not be possible to remove parental distress completely from what is an upsetting time. However, it is important for staff to communicate NBS+ results in a manner that minimises distress to families and does not detrimentally affect parents’ relationships with their child and other family members. Empirical evidence is lacking on the potential impact of information provision on parental well-being and decision-making strategies. As finite budgets are available to provide communication strategies on a national level, there is a need to understand both the short and long-term costs of different aspects of the NBSP including the implications of providing NBS+ results. A further consideration is ensuring parents are informed well enough to facilitate communication within and between family members.

The aim of this study is to work with parents to co-design, implement and undertake a process evaluation (including cost analysis) of new interventions to improve the delivery of initial NBS+ results to parents.

## Methods

### Design

**Fig. 1:**
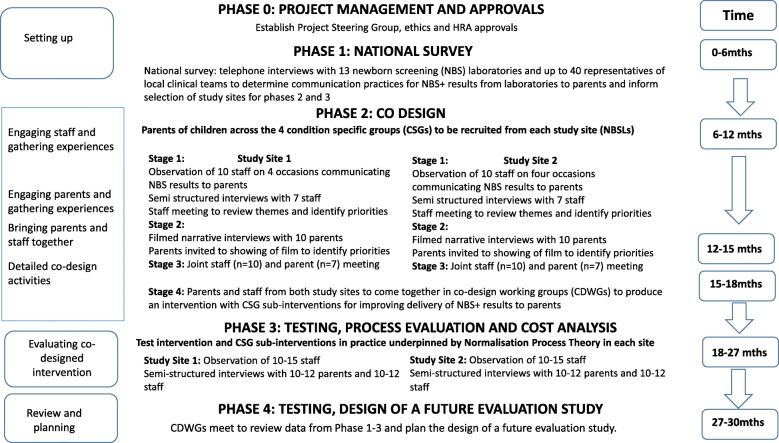
Study flow chart

The theory underpinning the proposed study is Family Systems Theory [16] because of the potential vulnerability of family relationships if the initial NBS+ information is not shared as effectively and empathetically as possible [17]. This mixed-methods study will use four phases with defined outputs and will be guided by the Medical Research Council Complex Interventions Framework [18]. A description of the phases and the study design can be seen in Fig. 1. The principles and methods of experience-based co-design (EBCD) will underpin intervention development [19–25]. Normalisation Process Theory [26, 27] will underpin the process evaluation of the new, co-designed interventions to improve the delivery of NBS+ results to parents. An economic analysis will be undertaken to determine resource use and costs of current practice and implementing the new co-designed interventions. The process evaluation will be used to explain discrepancies between expected and observed outcomes, contribute to understanding how context influences those outcomes and provide insights to aid planning of the future evaluation study. The economic analysis will provide additional data that will also be useful to stakeholders and decision makers [18]. The nominal group technique [28, 29] will be used to plan the future evaluation study including selection of suitable outcome measures.

The nine conditions currently screened for by the NBSP will be grouped into four condition-specific groups (CSGs) based on the urgency with which communication of the NBS+ result should occur (Table 1).

**Table 1:** Condition-specific groups (CSGs)

Group	Urgency	Conditions
Genetic/metabolic	Urgent—at immediate risk	Medium-chain acyl-CoA dehydrogenase deficiency, maple syrup urine disease, isovaleric aciduria
Genetic/metabolic	Urgent—not at immediate risk	Sickle cell disorder (SCD), cystic fibrosis (CF), phenylketonuria, homocystinuria and glutaric aciduria type 1
Other affected	Important—not at immediate risk	Hypothyroidism
Carriers	Non-urgent	SCD, CF

### Setting

In England, there are 13 newborn screening laboratories that process the results for the nine conditions that are currently included in the NBSP, and these will comprise the study sites.

### Inclusion and exclusion criteria

For all study phases, parents of children who have received a NBS+ result in the previous 3–12 months including true positives, false positives and children who are diagnosed as ‘cystic fibrosis screen positive, inconclusive diagnosis’ will be included in the study. Parents of children who have received a negative NBS result or with children with co-morbidities that are likely to influence their perception of receiving their NBS+ result and parents whose recruitment is contraindicated on psychosocial grounds (identified by their health visitor or specialist nurse) will be excluded. Parents who are unable to understand and give informed consent will also be excluded.

For all study phases, staff employed in NBS laboratories and involved in the processing of NBS+ results and staff who have been involved in communicating NBS+ results to parents in the last 6 months will be included. Staff who have not been involved in processing NBS+ results or communicating NBS+ results to parents in the last 6 months or who have personal experience of receiving a NBS+ result will be excluded.

### Recruitment

For phase 1, contact details of Directors of NBS laboratories will be identified through the relevant website^2^. Directors of newborn screening laboratories will be invited to be the Lead Investigator for the study site and will be asked to provide names and contact details of staff within the laboratory who meet the inclusion criteria for the study. These staff members will be contacted via email and invited to participate. Members of relevant clinical teams will be identified through the individual trust websites.

During phases 2 and 3, staff identified in phase 1 as being involved in the communication of positive newborn screening results to parents in the selected study sites will be contacted via email and invited to participate in these phase of the study.

During phases 2 and 3, parents who fit the inclusion criteria will be identified by the relevant clinical nurse specialist. Once eligible parents have been identified, a member of the clinical team (Clinical Nurse Specialist or doctor) will provide the parent with a participant information sheet at their next routine clinic appointment and ask the parents’ permission to provide their name and telephone number to a member of the research team. At least 24 h later, a member of the research team will telephone the parents, give them the opportunity to ask questions about the study and ask if they wish to proceed with being involved.

During phase 4, key stakeholders will be identified by the study steering committee using purposive sampling to ensure the four CSGs are represented.

#### Demographic data

For all parents recruited to the study, data regarding their age, gender and ethnicity will be collected. In addition, they will be asked if the child who has received the NBS+ result is their first child and whether they have any other children. If they have other children, the ages of these children as well as whether or not they also have any long-term medical conditions will also be ascertained.

#### Consent

Written informed consent will be gathered for all participants for each phase.

### Phase 1: National survey

A national survey will be conducted to identify examples of current approaches, and associated resource use, for communication of NBS+ results from all 13 NBS laboratories via clinical teams to parents for each CSG in England. The survey will be informed by the literature and piloted before use in the main study. The survey, comprising closed and open-ended questions, will be conducted using semi-structured telephone interviews. It will identify the ways NBS+ results are communicated from the NBS laboratories to parents via a range of health professionals by collecting data on the following: the mode of communication strategy (face-to-face, letter, telephone, e-mail), the resources involved in each communication strategy, who provides the information and their role, and location (co-located or alternative site) of relevant services for the CSG. The communication pathway currently used will be identified from the point at which the laboratory produces the test result to when the parents are told the definitive result.

#### Participants

Directors of all 13 NBS laboratories in England will be invited to participate. In addition, up to 40 representative members (10 for each CSG) of local clinical teams (medical consultants, general paediatricians, nurse specialists, health visitors, specialist screening nurses, genetic counsellors) who receive laboratory results and are identified as being involved in communicating positive newborn screening results to parents will be invited as well.

#### Data analysis

**Fig. 2:**
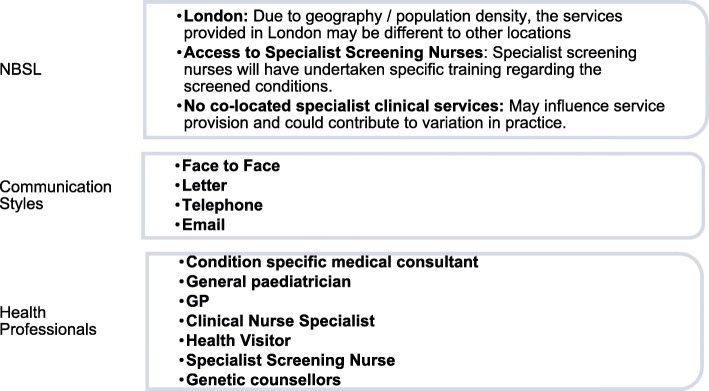
Exemplar sampling framework: features of the communication process for NBS+ results to parents

The purpose of data analysis will be to describe and identify approaches currently used to communicate the NBS+ results and identify potential study sites for phase 2 using a predefined sampling framework (Fig. 2). Quantitative data collected from the closed-ended questions will be analysed using descriptive statistics. This might include, for example, the number of people involved for each CSG, the median and range of times each NBS laboratory has been processing NBS results and the median and range of times taken when communicating a NBS+ result to clinical teams. Qualitative data from the open-ended questions will be analysed using content analysis [30]. An inductive approach will be adopted. One interview transcript will be coded separately by two members of the research team (double coded) in order to aid coding comparisons and inform and align code development [31]. A code book will be developed based on these comparisons and subsequent discussions in order to define the codes to be used. This will be an ongoing, iterative process; new codes may be developed during data analysis and the definition of codes refined as analysis progresses [32] Communication pathways for each of the 13 NBS laboratories (unit of analysis) will be described, by combining quantitative and quantitative data. These data will be presented to members of the study steering committee and the lay advisory group who will be involved in the decision regarding which study sites will be used in subsequent study phases. These decisions will be based on data from phase 1 regarding communication strategies currently in use, statistics regarding the number of babies who receive NBS+ results in each of the NBS laboratories on an annual basis [2, 3] and the sampling framework (Fig. 2).

Data collected during this phase will also be used in phase 3 to determine the total cost of existing communication strategies, from the NHS perspective by determining the grade of the person involved in the communication, the time taken and resources used.

#### Output(s)

The outputs are the following: (i) report describing current communication practices and (ii) selection of relevant study sites for phases 2–3.

### Phase 2: Co-design

This phase will consist of implementing the EBCD approach [21, 24] in the two study sites selected in phase 1 and will be guided by the EBCD Toolkit^3^. EBCD is an approach to improving healthcare services that draws on participatory design and user experience to bring about quality improvements in healthcare organisations [23]. EBCD involves focussing on and designing patient/carer experiences rather than just systems and processes [19, 24, 25] and—through a ‘co-design’ process—enables staff, patients and carers to reflect on their shared experiences of a service and then work together to identify improvement priorities, devise and implement changes, and then jointly reflect on their achievements.

In line with the EBCD approach, this phase will include non-participant observation of staff communicating the NBS+ result to parents. When the relevant member of the clinical team contacts the family to communicate the initial NBS+ result (by whichever methods they normally use, e.g. phone or face-to-face), they will ask the family at the beginning of the interaction, whether a member of the research team may be present. If the family agree, a researcher will observe the clinician communicating the NBS+ result to the family. During the communication, the researcher will not participate in the interaction between the clinician and the family but will take detailed field notes. Semi-structured interviews with staff to explore their experiences of communicating NBS+ to families and then a staff event to review themes that have arisen and identify priorities going forward will also be undertaken. Alongside this, filmed narrative interviews with parents of children who have received a positive NBS result will be conducted. This will be followed by a parent event where a composite film of the narrative interviews will be viewed and emerging issues and priorities identified. Following the separate staff and parent events, a mixed staff and parent event will be held at each site. These will enable joint priorities for improving delivery of NBS+ results to be developed using issues highlighted in the film and priorities from the separate staff and parent meetings. The joint priorities will guide the four co-design working groups who will meet on three occasions each. During co-design working group meetings, parents and staff will work together to co-design the new interventions based on the jointly identified priorities and following the EBCD toolkit^3^.

#### Participants

Samples sizes for this phase are based on previous successful EBCD studies [20–25]. A purposive sample of 15 staff representing the four CSGs delivering NBS+ results to parents across the two study sites will be the subjects for the non-participant observation. These staff will be interviewed about their experiences and invited to the staff event.

Narrative interviews will be conducted with a purposive sample of 20 parents representing the four CSGs who have received a NBS+ result for their child during the previous 3–12 months. All parents interviewed will be invited to the parent event.

The 15 staff and the 20 parents who have been interviewed will be invited to participate in the joint staff and parent event and the co-design working groups. As per the EBCD toolkit^3^, if retention of parents or staff is problematic at this stage, new participants will be recruited for the co-design work.

#### Data analysis

Data from observation and interviews with staff will be pseudonymised; each participant will be allocated a study code. Data obtained during observation of staff will be analysed using an inductive approach and themes will be generated using a manifest/semantic approach [33] in order to provide a rich description of current practice.

Staff interviews will be audio recorded and transcribed verbatim and will be analysed for themes [33] to inform the joint staff/parent meeting and subsequently the co-design working groups (CDWGs). An inductive approach to data analysis will be used and themes will be generated using a latent approach [33] to provide a deeper understanding of approaches used to communicate positive NBS results to families. The six phases of thematic analysis described by Braun and Clarke [33] will guide data analysis. One interview for each of the CSGs will be coded separately by two members of the research team (double coded) using NVivo software. This will aid coding comparisons and inform and align code development [31]. A code book will be developed based on these comparisons and subsequent discussions in order to define the codes to be used. This will be an ongoing, iterative process; new codes may be developed during data analysis and the definition of codes refined as analysis progresses [32]. Collating codes into potential themes and reviewing and defining the themes will be undertaken jointly by the two members of the research team who generated the initial codes [31, 33]. Themes identified from parent interviews will be made into a 30-min composite film [20–25]. The joint staff/parent meeting will generate the priorities that will be focussed upon by the co-design working groups.

The four CDWGs consisting of parents and staff will work together on proposed solutions for the identified priorities^3^. After each CDWG meeting, ideas generated by the group will be amalgamated and developed into protocols to aid implementation during phase 3. At the next CDWG meeting, the proposed interventions will be presented to the group so they may continue to work together to refine their ideas. The final interventions in the form of protocols will be presented at the final CDWG meetings to determine if any further tweaks are necessary before they are implemented in phase 3 for testing, process evaluation and cost analysis.

#### Output(s)

The output is the co-designed interventions for the four CSGs developed during the co-designed working group meetings^3^.

### Phase 3: Testing, process evaluation and cost analysis

**Fig. 3:**
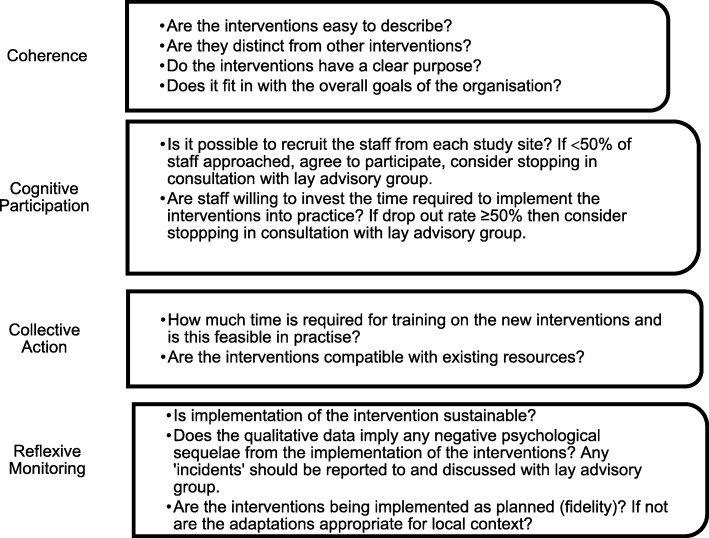
Success criteria for testing the co-designed interventions in routine practice

It is envisaged that the interventions will involve procedural changes that may include changes to documentation as well as staff training. The former will involve liaison between the research team, clinical teams and Public Health England, via the study support groups. Staff involved in the delivery of NBS+ results in the study sites will be trained to implement the new intervention protocols (developed as a result of the co-designed meetings) for the four CSGs concurrently. A training manual will be developed. Members of the research team will visit each study site and provide clinical teams for the CSGs with two face-to-face training sessions. Follow-up support will be provided including resource packs of information to support the use of the new co-designed interventions in practice, online resources made available to staff via a study-specific website and remote support via telephone/email. The face-to-face training will include a didactic approach but will also include the use of role play. Staff will be asked to evaluate the training to ensure it has met their needs and identify areas for improvement. Success criteria including the acceptability and feasibility of the co-designed interventions will be defined and monitored on a weekly basis during implementation (Fig. 3).

A parallel process evaluation [18] underpinned by Normalisation Process Theory [26, 27] will be conducted. Non-participant observation of staff delivering NBS+ results to parents and semi-structured interviews with parents and staff will identify healthcare resources required for delivery of the interventions, staff and parental experiences and factors that influence implementation [34, 35]. These qualitative data will also be used to determine suitable outcome measures for a future evaluation study.

An economic analysis will also be conducted. Our analytical approach will be to undertake a cost analysis of the intervention coupled with a feasibility study to plan the economic evaluation that would accompany a definitive evaluation study. The analytical approach that we envisage using in the definitive study would either be a cost-utility analysis or a cost-consequence analysis, and which of these will be most appropriate will be determined during the present feasibility study. The objectives are to (i) calculate the costs of the current and newly co-designed communication strategies (using data collected in phase 3 and phase 1) and (ii) inform the design of a feasibility study for an economic evaluation of options to improve delivery of initial NBS+ results to parents (in phase 4).

To meet the first objective, we will conduct an economic analysis to evaluate the NHS costs of the current and new communication strategies. This will include training costs, staff costs, and costs of consumables (e.g. information booklets). For training costs, we will include costs of the trainer time, training materials, training attendee numbers and time and any other costs associated with training that might be incurred, such as room hire. We will identify resource use for each of these items from training providers and value them using market prices. For staff costs and consumables associated with delivery of each communication strategy, we will produce flow charts for each strategy identifying the main activities and consumables. We will then produce a pro forma for each strategy and ask staff delivering the strategies to record the resources used on this pro forma which will be an electronic document that upon completion can be emailed directly to the study team. We envisage the main cost is likely to be staff time, so therefore, the pro forma will record the amount of time spent on different activities and the staff type and grade performing that activity. Staff time and consumables will be valued using published market prices. The main output will be a pictorial display (flow chart) of each strategy, resource use associated with that strategy and its cost.

For the second objective, we will plan the economic evaluation that would accompany a full evaluation study, identify potential sources of data and how best to collect these. Hence, the aim of the economic analysis in the present study is not to provide a definitive analysis of the costs, cost-effectiveness and budget impact of the planned interventions as that will not be possible until the full evaluation study (assuming this is shown to be feasible).

#### Participants

Thirty staff involved in delivery of NBS+ results will be trained to implement the co-designed interventions. These staff will be observed delivering NBS+ results to parents using the new co-designed interventions. Twenty parents who have received a NBS+ result in the previous 3–12 months and 20 staff who have communicated a NBS+ result to parents in the previous 12 months will be interviewed. Sample sizes are based on recommendations and findings regarding pilot and feasibility studies [36, 37] to demonstrate feasibility of recruitment, implementation and sustainability of the co-designed interventions in practice.

#### Data analysis

Qualitative data collected during the observation and semi-structured interviews will be used to identify factors that influence experiences during the delivery of NBS+ results. These data will be compared with the following validated scales: GAD-7 (generalized anxiety disorder), PHQ-9 (Patient Health Questionnaire), the Parenting Stress Index [38], EQ5D^4^ (a measure of health-related quality of life) and the ICECAP-A [39] (a measure of capability for the general adult population for use in economic evaluations) to determine where most overlap occurs to inform which outcomes might be most suitable in a future evaluation study.

Data from the non-participant observation will be analysed thematically [33] following the process described in phase 2. An inductive approach to data analysis will be used, and themes will be generated using a manifest and latent approach [33]. These might include the structures (processes) and use of healthcare resources (type and time) required for delivery of the interventions, how parents and staff respond (implementation and mechanism of impact) and how external factors (language barriers, cultural difference) influence implementation of the intervention(s) (context) [34, 35]. All interviews will be audio-recorded and transcribed. A deductive approach to thematic analysis will be used, and themes will be generated using both a manifest and latent approach as per phase 2 [33].

For the economic analysis, we will assess the costs of each communication strategy from an NHS perspective (i.e. calculate the costs to the NHS of delivering that strategy). Resource use data for each strategy will be collected as described above and combined with market prices (unit cost data form published and other sources) to calculate the total costs associated with each strategy.

#### Output(s)

The outputs are the following: (i) resource use and cost of current communication strategies (using data from phase 1) compared to resource use and costs associated with the co-designed interventions, (ii) acceptability and feasibility of the co-designed interventions based on the success criteria in Fig. 3, and (iii) choice of potential outcomes measures (GAD 7 PHQ 9 PSI [38] EQ5D^4^ and ICECAP-A [39]) for use in a future evaluation study.

### Phase 4: Design of future evaluation study

A meeting of key stakeholders (NBS co-ordinators, directors of NBS Laboratories, health visitors, midwives, genetic counsellors, parents) will be convened, and the nominal group technique (NGT) [28, 29] used to inform the design of an evaluation study of the co-designed interventions. The NGT was first developed by Delbeq and Van de Ven [40] and consists of a structured meeting consisting of steps during which participants generate, rate, discuss and then rerate a series of items or questions to achieve consensus regarding a given topic [28, 29]. In this study, adaptations to the NGT described in the literature [41–43] will be used during the initial round of idea generation in order to incorporate data collected during phases 1–3 of the study. An outline of the use of the NGT in the study can be seen in Table 2.

**Table 2:** The modified nominal group technique process

(i) Generating ideas	Members of the group will be asked to individually consider the data that has been presented to them and consider: 1. If there is there a need for an evaluation study of the co-designed interventions and if so; 2. Ideas for the potential design of an evaluation study of the co-designed interventions.
(ii) Recording ideas	Group members engage in a round-robin feedback session to concisely record each idea (without debate). If it becomes apparent that the consensus is not to proceed to an evaluation study, the NGT will cease at this stage. If it is decided that an evaluation study should be designed based on the data from phases 1–3, member of the group will be asked to share their ideas for the potential design of an evaluation study of the co-designed interventions.
(iii) Clarification	A discussion focussed on clarification of the ideas generated.
(iv) Voting	Individual group members vote privately to rank ideas. The votes will be tallied to identify the ideas that are rated highest by the group as a whole and will then be presented back to the group.
(v) Discussion	A group discussion to provide further clarification of the highest rated ideas for the future evaluation study of the co-designed interventions.
(vi) Re-ranking	Re-ranking of ideas to determine priorities and future plans.

#### Participants

A purposive sample of staff and parents (*n* = 10) involved in phase 2 as well as representatives from relevant charities and members of the research team will be invited.

#### Data analysis

Qualitative data collated during the NGT will be analysed using thematic analysis [33]. A deductive approach will be employed, and themes will be generated using a manifest/sematic approach in order to stay close to the data. The process described by Braun and Clarke [33] will be followed as described in phase 2, but data will be jointly coded by two members of the research team using NVivo software to ensure reliability of coding [31]. Quantitative data, such as ranking or rating data, will be summarised using descriptive statistics.

#### Outcome(s)

The outcomes are the following: (i) need for and design of a future evaluation study which may be condition-dependent and (ii) choice of relevant outcome measures.

### Dissemination

We intend to share our study findings on the national NBS websites so that they may be available to relevant health professionals involved in the delivery of the initial positive NBS result. We also plan to share our findings on the websites of the relevant charities and support groups associated with these conditions all of whom have been contacted and provided their endorsement for this study (CF Trust, Sickle Cell Society, British Thyroid Foundation, National Society for Phenylketonuria, Metabolic Support UK). We also aim to share our findings at relevant conferences nationally and internationally. We will submit our findings to high impact, peer-reviewed journals including the NIHR HS&DR journal. Parents involved in the study and those who form the advisory group will also be sent a summary of the research findings.

## Discussion

This protocol paper describes a national (England) study to develop co-designed interventions for the communication of NBS+ results to parents.

An important consideration when designing this study was the ethical issues associated with researching such a sensitive topic, i.e. communication of information to parents regarding life-changing/limiting conditions. Asking parents to recount the moment they received their child’s positive newborn screening result could be distressing for parents. However, the research team is highly experienced at working with families in this situation and will always proceed with due care and sensitivity. A member of the research team is a Consultant Clinical Psychologist and will be able to assist and advise should a parent become distressed during the interviews. At the end of each interview and parent/staff meeting and CDWG meetings, participants will be debriefed. Parents will also be offered options regarding where they would prefer the interviews to be conducted to minimise intrusion.

This study will lead to the development of co-designed interventions that will meet the needs of parents and health care professionals and will aim to minimise negative sequelae associated with communication of NBS+ results. This study will also involve the calculation and comparison of costs associated with different communication strategies as well as subsequent use of healthcare resources. This will include comparisons between costs of different approaches currently used (from phase 1) and costs of the new, co-designed interventions in terms of grade of staff involved, time taken and resources used (phase 3).

The proposed research may lead to the development of general evidence-based principles for communicating positive screening results for children and breaking bad news. This latter might include conditions that may or may not be life-altering/threatening but nevertheless can be distressing for parents, for example, newborn hearing screening, physical examination of newborn babies including congenital cardiac abnormalities, congenital cataracts, cryptorchidism, developmental dislocation of the hip and findings from screening of children’s eyes. It may also be possible to extrapolate findings from the present study for the delivery of bad news to parents for children newly diagnosed with cancer or following diagnosis of chronic conditions such as diabetes or epilepsy.

In order to monitor the success and progress of the study, an independent study steering committee, a project advisory group and a lay advisory group will be convened at the start of the study and will meet in person every 6 months for the duration of the study. The research team will also liaise via telephone conference call monthly for the duration of the study.

The study steering committee will consist of an independent Chair, external stakeholders (such as representatives from Public Health England and relevant charities), relevant methodologists (such as a health economist) and a clinician. The purpose of this committee will be to provide advice on aspects of the study to stakeholders, monitor the progress of the study, ensure the rights, well-being and safety of participants are maintained, ensure appropriate ethical and other approvals are obtained and agree substantial protocol amendments.

## Data Availability

Not applicable.

## Abbreviations

CDWG: Co-design working group
CF: Cystic fibrosis
CSG: Condition-specific group
EBCD: Experience-based co-design
EQ5D: Euro Qol 5D
GAD: Generalised Anxiety Disorder Assessment
ICECAP-A: ICEpop CAPability measure for Adults
NBS: Newborn blood spot screening
NBS+: Newborn blood spot screen positive
NBSP: Newborn blood spot screening programme
NGT: Nominal group technique
PHQ: Patient Health Questionnaire
SCD: Sickle cell disease

## Footnotes

1
https://www.gov.uk/government/collections/newborn-blood-spot-screening-programme-supporting-publications

2
http://newbornscreening.org/site/index.asp

3
https://www.kingsfund.org.uk/projects/ebcd

4
http://www.euroqol.org/home.html
